# Phenotypic variability in ARCA2 and identification of a core ataxic phenotype with slow progression

**DOI:** 10.1186/1750-1172-8-173

**Published:** 2013-10-28

**Authors:** Cyril Mignot, Emmanuelle Apartis, Alexandra Durr, Charles Marques Lourenço, Perrine Charles, David Devos, Caroline Moreau, Pascale de Lonlay, Nathalie Drouot, Lydie Burglen, Nadine Kempf, Elsa Nourisson, Sandra Chantot-Bastaraud, Anne-Sophie Lebre, Marlène Rio, Yves Chaix, Eric Bieth, Emmanuel Roze, Isabelle Bonnet, Sandrine Canaple, Coralie Rastel, Alexis Brice, Agnès Rötig, Isabelle Desguerre, Christine Tranchant, Michel Koenig, Mathieu Anheim

**Affiliations:** 1 Department of Genetics and Cytogenetics, AP-HP, Hôpital de la Salpêtrière, Paris, F-75013, France; 2 Service de Neurologie Pédiatrique, APHP, Hôpital Armand Trousseau, Paris, France; 3 Centre de Référence des Déficiences Intellectuelles de Causes Rares, Paris, France; 4 Groupe de Recherche Clinique (GRC) 'déficience intellectuelle et autisme’, UPMC Univ Paris 06, Paris, France; 5 Department of Physiology, APHP, Saint-Antoine Hospital, Paris, France; 6 Physiopathology and Treatment of Neurodegenerative Disorders, Inserm, UMR_S975, CRICM, Team Molecular Bases, Paris, F-75013, France; 7 UPMC Univ Paris 06, UMR_S975, Paris, F-75013, France; 8 CNRS UMR 7225, Paris, F-75013, France; 9 Department of Neuroscience and Behaviour Sciences, School of Medicine of Ribeirão Preto, University of Sao Polo, Sao Polo, Brazil; 10 Department of Medical Pharmacology & Department of Movement Disorders and Neurology, EA 4559/1046, Lille Nord de France University, CHU Lille, Lille, France; 11 Department of Movement Disorders and Neurology, EA 4559/1046, Lille Nord de France University, CHU Lille, Lille, France; 12 Inserm U781, Imagine Institut des Maladies Génétiques, Université Paris Descartes et Centre de Référence des Maladies Héréditaires du Métabolisme, Hôpital Necker, AP-HP, Paris, France; 13 Institut de Génétique et de Biologie Moléculaire et Cellulaire (IGBMC), INSERM-U964/CNRS-UMR7104/Université de Strasbourg, Illkirch, 67404, France; 14 Service de Génétique et d’Embryologie Médicale, AP-HP, Hôpital Armand Trousseau, Paris, France; 15 Centre de Référence des malformations et maladies congénitales du cervelet, Paris, France; 16 Laboratoire de Diagnostic Génétique, Hôpitaux Universitaires de Strasbourg - Nouvel Hôpital Civil, Strasbourg, France; 17 Université Paris Descartes; INSERM U781, Assistance Publique-Hôpitaux de Paris - Hôpital Necker-Enfants Malades, Paris, 75015, France; 18 Département de Génétique, Hôpital Necker-Enfants Malades, Assistance Publique-Hôpitaux de Paris, Paris, France; 19 Unité de Neurologie Pédiatrique, Hôpital des Enfants, Toulouse, France; INSERM U825, Hôpital de Purpan Toulouse, Toulouse, France; 20 Service de Génétique, Hôpital Purpan, Toulouse, France; 21 Département de Neurologie, AP-HP, Groupe Hospitalier Pitié-Salpêtrière, Paris, France; 22 Cabinet de Neurologie, Amiens, France; 23 Laboratoire de Neurosciences Fonctionnelles et Pathologies, Service de Neurologie, CHU Amiens, 80054 Amiens Cedex, Amiens, EA4559, France; 24 Institut Imagine and INSERM U781, Université Paris Descartes-Sorbonne Paris Cité, Paris, France; 25 Service de Neurologie Pédiatrique, APHP, Hôpital Necker-Enfants Malades, Paris, France; 26 Département de Neurologie, Hôpitaux Universitaires de Strasbourg, Hôpital de Hautepierre, Strasbourg cedex, 67098, France; 27 Fédération de Médecine Translationnelle de Strasbourg (FMTS), Université de Strasbourg (UdS), Strasbourg, France

**Keywords:** Cerebellar ataxia, Genes, Recessive, Movement disorder, Seizure disorder, Mitochondrial disorders

## Abstract

Autosomal recessive cerebellar ataxia 2 (ARCA2) is a recently identified recessive ataxia due to ubiquinone deficiency and biallelic mutations in the *ADCK3 gene*. The phenotype of the twenty-one patients reported worldwide varies greatly. Thus, it is difficult to decide which ataxic patients are good candidates for *ADCK3* screening without evidence of ubiquinone deficiency. We report here the clinical and molecular data of 10 newly diagnosed patients from seven families and update the disease history of four additional patients reported in previous articles to delineate the clinical spectrum of ARCA2 phenotype and to provide a guide to the molecular diagnosis. First signs occurred before adulthood in all 14 patients. Cerebellar atrophy appeared in all instances. The progressivity and severity of ataxia varied greatly, but no patients had the typical inexorable ataxic course that characterizes other childhood-onset recessive ataxias. The ataxia was frequently associated with other neurological signs. Importantly, stroke-like episodes contributed to significant deterioration of the neurological status in two patients. Ubidecarenone therapy markedly improved the movement disorders, including ataxia, in two other patients. The 7 novel *ADCK3* mutations found in the 10 new patients were two missense and five truncating mutations. There was no apparent correlation between the genotype and the phenotype. Our series reveals that the clinical spectrum of ARCA2 encompasses a range of ataxic phenotypes. On one end, it may manifest as a pure ataxia with very slow progressivity and, on the other end, as a severe infantile encephalopathy with cerebellar atrophy. The phenotype of most patients, however, lies in between. It is characterized by a very slowly progressive or apparently stable ataxia associated with other signs of central nervous system involvement. We suggest undergoing the molecular analysis of *ADCK3* in patients with this phenotype and in those with cerebellar atrophy and a stroke-like episode. The diagnosis of patients with a severe ARCA2 phenotype may also be performed on the basis of biological data, i.e. low ubiquinone level or functional evidence of ubiquinone deficiency. This diagnosis is crucial since the neurological status of some patients may be improved by ubiquinone therapy.

## Introduction

Autosomal recessive cerebellar ataxias (ARCAs) are a group of neurodegenerative disorders defined by a persistent and gradually worsening disorder of gait and balance or with the development over months or years of hypotonia or excessive clumsiness [1]. In most cases, ARCAs start in childhood or adolescence and lead to a loss of ambulation within 10-20 years. ARCA2 is a recently identified entity reported in 21 patients worldwide [2-6]. It is due to recessive mutations in the *ADCK3*/*CABC1* gene leading to coenzyme Q10 (CoQ10) deficiency, and is also known as COQ10D4 and SCAR9 (spinocerebellar ataxia recessive 9, MIM #612016). Given the genetic heterogeneity of ARCAs, the identification of key clinical features of ARCA2 is of particular importance for physicians facing the diagnosis of an ataxic individual. If the parents of the patient are healthy, the hypothesis of a recessive ataxia is reasonable and molecular studies will be guided by clinical, radiological and biological findings. On the other hand, if variants of unknown significance are identified in *ADCK3* with new generation sequencing analyses, the diagnostic process will be guided by their clinical relevance.

Previous articles showed that the ARCA2 phenotype varied greatly from one patient to another. Indeed, the neurological involvement appeared either as a pure ataxia [4], or as progressive ataxia with cerebellar atrophy in addition to intellectual deficiency, epilepsy, stroke-like episodes (SLE) and/or exercise intolerance in some patients [4-7], or as a severe and progressive encephalopathy comprising cerebellar atrophy in others [5]. Furthermore, the ataxia seemed to be progressive in some patients and apparently self-limited in others [2]. Thus, the ARCA2 phenotype remains difficult to define. Radiological data did not provide any clue to the diagnosis, except that stroke-like images may be observed [5]. The relevance of biological data is limited by the conflicting results of routine assays (lactacidemias, ubiquinone assays), but muscular ubiquinone deficiency and mitochondrial respiratory chain assessment seem to provide reliable clues to the diagnosis [4-6]. Thus, to date, the decision to screen *ADCK3* mutations mainly relies on the evidence of muscular ubiquinone deficiency.

We report the clinical history and molecular findings in 10 new ARCA2 patients and provide detailed and updated clinical data of four other patients previously reported [4,5]. We compare our data with those of the literature in an attempt to facilitate the decision for *ADCK3* molecular study and its interpretation in ataxic patients.

## Material and methods

### Patients

Since 2008, 419 patients affected by ataxia of unknown etiology with cerebellar atrophy whose DNA was sent to the diagnostic center were screened for *ADCK3* mutations (Dr Anheim and Dr Koenig). Biallelic mutations were found in nine patients (all but patient #7). Molecular diagnosis was made after ubiquinone deficiency was evidenced in patient #7 (Dr Rötig, Dr Lebre, Dr Desguerre, Dr Pascale de Lonlay). All samples were collected under informed consent. In addition on the ten patients, we retrospectively reassessed clinical data from four previously reported patients [4,5,7] and provide an update of the outcome thanks to their referral neurologists. Patient #11 has been reported as patient 7 in [4], patient #12 as patient 4 in [5,7], and patients #13 and #14 as patients 2 and 3, respectively, in [5].

Functional disability related to ataxia was evaluated with the Spinocerebellar Degeneration Functional Score [8,9] (SDFS: 0: no functional handicap; 1: no functional handicap but signs at examination; 2: mild, able to run; 3: moderate, unable to run; 4: severe, walking with one stick, unlimited walking; 5: walking with two sticks; 6: unable to walk, requiring a wheelchair; 7: bedridden). When possible, the clinical severity of ataxia was evaluated at last examination with the Scale for the Assessment and Rating of Ataxia (SARA) [10]. We used the Spearman rank correlation analysis to determine the association between the SDFS/ SARA scores and the duration of ataxia.

The Wechsler Intelligence Scale for Adults (WAIS) was used to evaluate the intellectual quotient in adult patients and the Wechsler Intelligence Scale for Children (WISC-IV) was used for patient #13.

### Movement disorders

Neurophysiological recording of movement disorders was performed with a Neuropack (Nihon Kohden, Japan) device. Movement was recorded with a unidirectional piezo-resistive accelerometer (Acc) (PCB Piezotronics, USA), Acc signal was band-pass filtered at 0.5–100 Hz. Electromyographic (EMG) signals were obtained from pairs of silver/silver chloride electrodes (Medtronic, Minneapolis, MN, USA) placed over muscle bellies, and band pass filtered at 20-500 Hz. The selection of recorded muscles was guided by the clinical location of tremor (upper limb and/or or neck).Tremor was recorded during rest, posture and action/intention. The effect of mental calculation was tested at rest. Postural and action/intention tremor were analysed during tonic contraction, slow elementary non goal-directed movements, goal-directed movements and spiral drawing. Stimulus-sensitive myoclonus was searched for with a slight distal touch, a pinprick and passive mobilization of the wrist. Dystonic features, namely co-contraction, overflow, spasms and asymmetries were carefully evaluated. The frequency of tremor and the bursts duration were measured by manual analysis of EMG and Acc epochs.

### Molecular genetics

The 14 coding exons and the adjacent intronic junctions of the human *ADCK3* gene were amplified and sequenced as detailed previously [4]. Sequences were analysed using the Seqpilot software version 2.0 (JSI medisys, Kippenheim, Germany) or the Seqscape 2.5 software (Applied Biosystems). Conservation of amino-acids involved in missense mutations was analysed with the blastp software of the NCBI platform ( http://blast.ncbi.nlm.nih.gov/Blast.cgi) with the non-redundant protein sequences (nr) database. The splice site mutation was analysed with the SplicePort software ( http://spliceport.cbcb.umd.edu/) and by RT-PCR as detailed previously [4] using primers located in exons 3 and 8 (forward 5′- TCTTTGCAAACCCCAGAGAC -3′, reverse 5′- CCCAGGTCGTTGTTGAGAGT -3′).

Partial gene deletion was assessed with the Illumina CytoSNP-12 arrays which contain 300,000 markers including 200,000 SNPs according to the manufacturer’s specifications (Illumina, San Diego, California, CA, USA). Image data were analysed with the Chromosome Viewer tool contained in Genome StudioV2001.1. The metrics used were the Log R ratio (in ratio of the sample copy number/reference copy number) and the B allele frequency (BAF) (the genotype of each SNP). Genomic positions were based on GRCh37/hg19 assembly of the UCSC genome browser ( http://genome.ucsc.edu).

## Results

### General data

The 10 new patients with *ADCK3* mutations identified since the first report were six females and four males, including six individuals from three unrelated sibships and four sporadic cases. Nine over 14 ARCA2 patients reported here were adults at the time diagnosis, three were adolescents and two were children. Nine mutations were found, including five homozygous and seven novel mutations. Clinical and molecular data of the 14 patients are available in Additional file 1: Table S1. A detailed description of representative clinical histories (patients #3, #5, #6 and #7) is available online as Additional file 2.

### ARCA2 onset

First clinical manifestations occurred at a mean age of 6.7 years (range 1.5-19 years), with obvious signs before or at 15 years in 11/13 patients. Gait ataxia was the first clinical manifestation in 8/14 patients at a mean age of 10.1 years (range 2.5-15 years). Cerebellar ataxia was obvious after the age of walking in all patients but one who never walked (patient #7). In this patient, truncal ataxia was rapidly associated with global developmental regression.

Clinical signs in the upper limbs were prominent at disease onset in 7/14 patients (mean age 5 years, range 2-15 years) in the form of clumsiness of hands, writing difficulties, hand dystonia or chorea. Obviously, the origin of some of signs may have been cerebellar, particularly when associated with gait ataxia. In other cases (4/7 patients), they preceded gait ataxia by 3 to 7 years.

Severe exercise intolerance was the first sign of ARCA2 in patient #12 (previously reported in [7]).

### The course of ataxia

**Figure 1:**
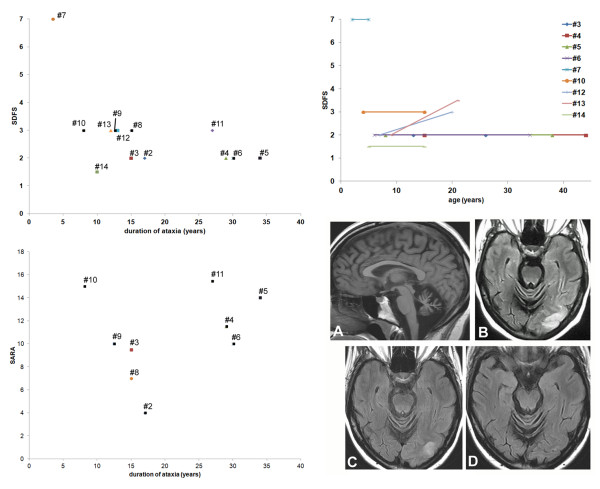
**Disability scoring and stroke-like lesions in patients with ARCA2.** Functional Score (SDFS, top left) and Scale for the Assessment and Rating of Ataxia (SARA, bottom left) evaluated during the last examination are presented according to the duration of ataxia in 13 and 9 patients (numbered #1 to #14), respectively. Patient #7 with infantile global brain degeneration clearly stands out from other patients with the mild phenotype (top left). SDFS and SARA scores do not appear to be correlated with the duration of ataxia, as confirmed by the Spearman rank correlation analysis (see text). The evolution of the SDFS with time was determined in nine patients (top right). It did not change during childhood, from childhood to adulthood and throughout adulthood in 7/9 patients. Patients #12 is the unique patient with muscular involvement, which may explain the worsening of the SDFS score with time. The neurological status of patient #13 worsened after a SLE, and then partially recovered. Bottom right subpanel: brain MR images of patient #5 showed vermian atrophy (**A**, T1-weighted image) and stroke-like lesions of the occipital lobe during the episode (**B**, FLAIR sequence). These images were attenuated one week after **(C)** the episode and no longer visible seven weeks later **(D)**.

In our patients series, mean age at last examination was 26.2 years (range 5-44 years) with mean disease duration of 19.4 years (range 3.5-34 years). Excluding patient #7 who never walked, mean duration of ataxia for the 13 other patients was 18.5 years (range 8-34 years, median 15 years). All these patients were ambulatory except patient #13 who had to use a walking aid after a SLE. The semi-quantitative SARA score was assessed during the last examination for 10 patients (Additional file 1: Table S1), ranging from 4 to 15.5/40 (mean 10.7, standard deviation 3.8). SARA scores were not apparently correlated with the age of ataxia onset and with the duration of ataxia when known (9/10) (Figure 1, bottom left), which was confirmed by the Spearman rank correlation analysis (rho values -0.23 and -0.15, respectively). Likewise, the functional scores were 1.5 to 7 (mean SDFS 2.8), irrespectively of the duration of ataxia (Figure 1, top left, rho values -0.13 for ataxia onset and -0.21 for ataxia duration). The patients, their parents and/or their pediatric medical records allowed a retrospective evaluation of the functional score for nine of them (Figure 1, top right). It mildly worsened over a period of 12 to 29 years in two patients, which may be partially explained by the concomitant muscular involvement in patient #12 and by the occurrence of a SLE in patient #13 [5]. In patient #7 the severity of functional outcome was extreme from the onset. In five other patients, the functional score was remarkably stable and mild over a mean period of 18.6 years (range 10-30 years).

### Patients with a severe neurological phenotype

Patient #7 with severe neurodegeneration from infancy is the only patient of our series with this phenotype. All ARCA2 features reported in other patients with a mild severity (epilepsy, dystonia, pyramidal syndrome, intellectual deficiency) were present in patient #7 but with a much more severe intensity. Her brain MRI disclosed global brain atrophy, not only cerebellar atrophy.

Patients #5 and #13 [5] had a single SLE at the ages of 34 and 14 years, respectively. In both cases, it presented as a status epilepticus with residual epilepsy and deterioration of intellectual and motor functions in patient #13, and with residual partial visual loss in patient #5.

### Clinical features associated with ataxia

#### Movement disorders and polymyographic recordings

Movement disorders unrelated to the cerebellar syndrome were present in 9/14 patients of our series and sometimes combined in the same patient: myoclonus in 6/14, dystonia in 6/14, chorea in 2/14 and tremor in 2/14 (Additional file 1: Table S1). In one patient, unilateral dystonia was as the consequence of a SLE (patient #13). In the others, hand or neck dystonia was mild, could be fluctuating, clinically significant in daily life, and reportedly stable with time. Patient #10 had hand chorea from the age of 2 to 7 years, the age at which gait ataxia appeared.

**Figure 2:**
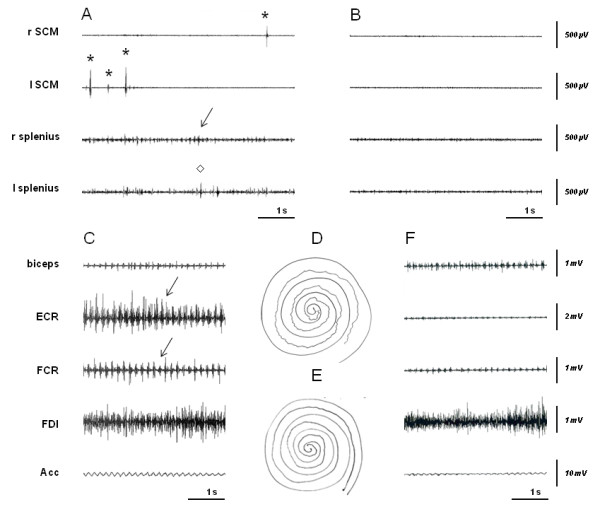
**Polymyographic recordings of tremor and myoclonus in two patients with ARCA2 demonstrating the effect of ubidecarenone therapy.** Patient #3: note the erratic myoclonus (*) obtained in SCM muscles (90 ms), during writing **(A)**. Dystonic tremor (arrow), can be seen in posterior muscles (right and left splenius), intermingled with myoclonus (◊). All these abnormalities were absent after treatment **(B)**. Patient #4: left upper limb recording performed during spiral drawing displayed a 6 Hz regular tremor (arrow), composed of 120 ms length bursts, alternating in antagonistic forearm muscles (ECR/FCR), associated to an oscillatory signal in Acc **(C)**. Note the dramatic attenuation of tremor amplitude after treatment on spiral drawing (**D**, before treatment and **E**, under ubidecarenone), as well as on polymyographic recording (**F**, under treatment). Abbreviations: r: right, l: left, SCM: sterno-cleido-mastoideous, ECR: extensor carpi radialis, FCR: flexor carpi radialis, FDI: first dorsal interosseous muscles, Acc: accelerometer.

Erratic myoclonus were observed in 6/14 patients and tremor in three. Myoclonic jerks were of low amplitude but obvious at examination. Polymyographic recording disclosed mild myoclonus and tremor in neck muscles suggesting dystonic tremor in patient #3 (Figure 2A), and myoclonus associated with mild hand dystonia in patient #12. Here, myoclonic jerks were absent at rest, not elicited by tactile or pinprick distal stimuli nor worsened with action, had no peculiar temporo-spatial organization. They were longer (80-135 ms) than expected for a cortical generator. No abnormalities were found in patients #5 and #6 though dystonia and tremor were clinically diagnosed a few hours after recording, which was in line with the reported fluctuations. A regular postural/action tremor (frequency 6 Hz, bursts duration 110 ms) was recorded in the upper limbs in patient #4 (Figure 2C).

#### Corticospinal tract involvement

Most patients had brisk tendon reflexes (11/14), two had a unilateral or bilateral plantar extensor reflex. Spasticity was disclosed only in patients #7 and #13, i.e. in one patient with severe brain degeneration and another with a SLE (hemiparesis in her case). The pyramidal syndrome did not appear to have functional consequences in other examined patients of our series.

#### Seizures/epilepsy and EEG

7/14 patients experienced seizures. Patient #12 had a single episode in childhood and never received any anti-epileptic drug (AED). Three patients had few seizures before the age of 20 years and received an AED without seizure relapse. AED have been successfully withdrawn in two of them. The two patients with a SLE with epileptic manifestations previously had well-controlled epilepsy. Patient #7 had severe drug-resistant epilepsy.

Though most seizures were reported as generalized tonic-clonic, interictal EEG recordings showed focal (4/8) or multifocal (1/8) spikes and/or spike-waves. The background activity was altered in patient #7 only.

#### Early development and intellectual functioning

Most patients (9/13) had a normal initial development during their first three years, i.e. acquired walking ability, language learning and an accurate use of hand in time. 3/13 had a mildly delayed acquisition of walking and/or a language delay. Finally, a global regression was observed at 18 months in patient #7 described above.

8/14 adult or adolescent patients were considered not to have an intellectual deficiency (ID), 5/14 to have mild ID, and patient #7 had an obvious profound ID. Formal evaluation in patients #3 and #4 confirmed normal intellectual functioning. Mild ID was confirmed in patients #11 (FSIQ 54, WAIS) and in patient #13 before the SLE (FSIQ 70, WISC-IV).

#### Muscular involvement

The only patient reported here with muscular involvement is patient #12 whose disease has been extensively detailed by others [7]. She is now 20 years, her muscular involvement is stabilized and she needs no walking aid.

### Other paraclinical tests

#### Brain MRI

In all patients, brain MRI disclosed cerebellar atrophy without cerebellar signal anomalies or any other specificity. It is not clear whether it was present from the onset of ataxia. The two patients with a SLE had MRI anomalies of the affected part of the cerebral cortex, i.e. of the parietal (reported in [5]) or occipital lobes (Figure 1, bottom right). Global brain atrophy was found in patient #7 with severe involvement.

#### Mitochondrial disease investigations

Plasma lactic acid levels were normal in 9/10 patients and increased in the patient with muscular involvement [7]. CSF lactic acid concentrations were normal in 8/8 patients. The assessment of mitochondrial oxidative phosphorylation showed normal enzyme activity in the muscle of patient #8 and a deficiency of complex II + III in patients #11 [7] and #7. Co-enzyme Q10 (CoQ10, ubiquinone) levels were normal in the fibroblasts of two patients and decreased in the blood of patient #7 and in the muscle of patient #11 [7]. Over the five patients who underwent a muscle biopsy, three had a normal pathological aspect and two had ragged-red fibres, i.e. patient #7 with severe involvement and patient #11 with muscular involvement.

### Molecular analysis of the *ADCK3* gene

All patients carried biallelic alterations of *ADCK3* (Additional file 1: Table S1) with a total of nine different mutations. Mutations inducing aminoacid substitutions were c.811C > T (p.Arg271Cys), c.895C > T (p.Arg299Trp), c.1523 T > C (p.Phe508Ser) and c.1844G > A (p.Gly615Asp). Truncating mutations were c.1081-1_1082dupGTA (p.Gln360_Tyr361ins*), c.1358delT (p.Leu453Argfs*24), c.1228C > T (p.Arg410*), c.589-3C > G (p.Leu197Valfs*20) and a 29 kb partial deletion of the gene including at least exons 3 to 15 (base pair position: 227,150,977-227,195,656, hg19).

The mutation c.1081-1_1082dupGTA is a 3-nucleotide duplication overlapping the acceptor splice site of exon 9. It does not alter the splice site but results in a 3-nucleotide insertion at the beginning of exon 9 which immediately creates an in-frame stop codon (p.Gln360_Tyr361ins*).

The homozygous c.589-3C > G mutation lying in intron 3 and found in sisters #8 and #9 affects the acceptor splice site of exon 4. It reduces the splicing score predicted with the SplicePort software from 3.02 to 1.53. *ADCK3* transcript analysis from the patients fibroblasts, who are homozygous for the mutation, indicates that usage of the normal exon 4 acceptor splice site is completely abolished and replaced by the usage of a cryptic splice site located 23 nucleotides upstream, causing a frame-shift and termination 20 codons after the last codon of exon 3 (p.Leu197Valfs*20). The SplicePort splicing score of the cryptic splice site is 2.11, explaining why it is preferably used over the mutant splice site but not over the normal splice site.

### Ubiquinone supplementation therapy

Twelve patients received ubiquinone supplementation therapy: 10 received ubidecarenone with doses varying from 100 mg × 3/day to 250 mg × 3/day and 30 mg/kg/d in patient #7, and two patients received idebenone at 45 mg × 3/day. No clear improvement was noticed in seven patients. In patient #12, the myoclonus was aggravated by idebenone. She recovered her previous status after its withdrawal and received ubidecarenone without clear improvement. Ubidecarenone had to be withdrawn in two patients who experienced reversible side effects (anorexia and diarrhea). The outcome is not known for one patient.

Two patients took advantage from ubidecarenone therapy. Patient #3 had a dramatic and long-lasting improvement of dystonia and myoclonus after six months of treatment confirmed by polymyographic recording (Figure 2B). His SARA score improved from 9.5 to 6.5. Likewise, the polymyographic recording of the tremor of patient #4 improved after 8 months, as well as his drawing ability (Figure 2E and 2F).

## Discussion

The description of the phenotype in the 14 patients reported here together with that of other patients of the literature (Additional file 3: Table S2) allows us to give an accurate picture of the ARCA2 from the first signs to adulthood and throughout adulthood.

### Variability of onset

First clinical manifestations of ARCA2 occurred at a mean age of 6.7 years in our series and at 19 years for the oldest patients. This is in line with the mean age of 6.6 years reported in 17 patients of the literature. Hence, ARCA2 mostly starts in childhood or adolescence, sometimes in early adulthood. The only exception (patient 2 in [3]) is a woman with adult-onset ataxia (46 years), but she experienced seizures in childhood, which may have been the first manifestation of ARCA2.

Like in our series, gait ataxia was the most frequent sign at disease onset in 11/14 patients of the literature, but difficulties with the use of hands were reported only twice [2,3]. Though the origin of hand clumsiness may have been cerebellar, particularly when associated with gait ataxia, this was not the case for all of our patients. Our series study suggests that dystonia or chorea of hands may be the first manifestations of ARCA2 in some patients. Other manifestations were rarer at disease onset, including exercise intolerance in patient #12 and epilepsy in three patients of the literature [3,6]. Taken together, these data indicate that the onset of ARCA2 is not stereotyped but manifests most often as early-onset ataxia, and sometimes as hand clumsiness or as epilepsy. Non-ataxic presentations may be misleading until ataxia or cerebellar atrophy is evidenced.

### Mild disease course in most patients

Since gait ataxia was the main criterion for *ADCK3* mutation screening, it was present in all patients reported to date, suggesting a possible screening bias. Despite phenotypical heterogeneity, it is noteworthy that all 10 affected sibs of seven probands reported so far also had gait ataxia, suggesting that cerebellar syndrome may be the common feature of all ARCA2 patients. Gait ataxia was associated with other cerebellar signs in the patients of our series as well as in most patients of the literature, when indicated, i.e. upper limb dysmetria, dysarthric speech and saccadic ocular pursuit.

Regarding the course of ataxia, the semi-quantitative evaluations of ataxia and disability gave strikingly mild scores in most patients, including in the elders. The slight progressivity or stability of ataxia in ARCA2 from childhood to adulthood and throughout adulthood has been previously suggested by other authors [2,3]. Indeed, data from the literature suggest a favorable ataxic outcome, since 14/17 patients were still ambulatory when reported after a mean ataxia history of 20.8 years (range 5-34 years, median 27.5 years). Among the three previously reported patients who became wheelchair bound, one had a SLE [5], like the significantly disabled patient #13 of our series, and two other had a global neurological regression from the ages of 1.5 and 3 years (patients 3 and 4 in [3]), like patient #7 reported here.

The mean SARA score in the 10 evaluated ARCA2 patients reported here is 10.7 after a mean duration of 19.4 years (ratio SARA/disease duration = 0.55 point/year). Most other genetic progressive ataxias are associated with a more unfavorable outcome. As an example, patients with Friedreich ataxia (whose first signs occur later than in ARCA2) have a mean SARA score of 22.7 ± 9 after mean disease duration of 17.3 ± 9.2 years (ratio 1.3 point/year) [11]. In ataxia with ocular apraxia type 2 (AOA2), the mean SDFS score is 4.7 after a mean disease duration of 18.9 years [12], giving a mean progression rate of the disability score (SDFS/disease duration) of 0.39 versus 0.19 for the 13 ARCA2 patients evaluated here. Moreover, in other ARCAs, deambulation is lost after a mean disease duration of 10 years in ataxia telangiectasia, 10-15 years in Friedreich ataxia and about 20 years in AOA2 [1], which contrasts with the 26/31 (83%) ambulatory ARCA2 patients affected for a mean duration of 18.6 years. Moreover, the slow evolution of ARCA2 ataxia is obvious when compared with dominant spinocerebellar ataxias (SCA) due to polyglutamine tract expansion in which the mean SARA score is 14.9 after a disease duration of 11 years (ratio 1.35 point/year) [13]. The mean SARA score of non polyglutamine-related SCAs is however closer to that of ARCA2, with a value of 11 after 16 years for (ratio 0.68 point/year) [13].

These data indicate that the progression of ataxia in ARCA2 is usually mild and that the neurological outcome in most ARCA2 patients is not an inexorable evolution towards loss of ambulation, unlike in most other ARCAs with childhood onset.

### At the other end of the spectrum, patients with a severe phenotype

In most patients, the unfavorable outcomes in ARCA2 patients of our series and of the literature were neither related to the worsening of cerebellar signs, nor to its association with spastic paraparesis or peripheral neuropathy, as it is the case in other ARCAs [1].

SLEs, which have been reported in several mitochondrial disorders [14,15], may explain the severity of the ARCA2 phenotype in 3/31 patients. SLEs were reported in two patients of the literature (both in [5], including patient #13 reported here) and occurred during adolescence as a single episode without relapse. In both cases, it presented as a status epilepticus with residual epilepsy and deterioration of intellectual and motor functions. Patient #5 reported in our series experienced a posterior SLE at the age of 34 years while her ataxia and epilepsy remained stable (Additional file 2). This suggests that some ARCA2 patients with an apparently stable disease may experience acute and disabling events at any age. In her particular case, the SLE could have been the consequence of a metabolic stress due to significant weight loss. While neurological functions declined after SLE in patients #5 and #13, the neurological involvement in their siblings was still limited and stable. This is consistent with SLE being an important factor that may explain the heterogeneity of the functional outcome among affected siblings.

The phenotype of patient #7 is more suggestive of an infantile degenerative encephalopathy rather than progressive ataxia. Given parental consanguinity, we could not exclude an associated genetic factor that could possibly explain this unique phenotype among the patients of our series. However, a similar phenotype has been reported by others (patients 3 and 4 in [3]), which confirms that early global neurological regression is the most extreme presentation of ARCA2. It is worth mentioning that ARCA2 in other patients with an onset before three years had a relatively mild disease course (family B in Gerards *et al.*, 2010), meaning that an early onset does not necessarily indicate a severe phenotype.

We conclude that, like in many other mitochondrial disorders, the severity of the neurological involvement in ARCA2 is variable, with about 19% of patients (6/31) being significantly disabled either because of a SLE or because their phenotype is a severe progressive encephalopathy rather than a degeneration of the cerebellum.

### ARCA2: a complex phenotype

Ataxia was the only sign of ARCA2 in 3/31 patients only (patient 1 of family 1 and patient 5 of family 2 in [4] and patient #2 reported here). Most patients, however, presented neurological signs in addition to ataxia.

Movement disorders (dystonia, myoclonus, tremor) were present in 9/14 patients our series and were also reported in previous articles: dystonia in four cases [2-4], myoclonus in three [2,3] and tremor in two [3]. Hand chorea observed in patient #10 from the age of 2 to 7 years is the only choreic ARCA2 patient reported to date and is a striking example of spontaneous neurological improvement with time. Six patients of our series had myoclonic jerks but they were reported in only three patients of the literature (total 9/31). Their frequency may have been underestimated since they were of low amplitude in most of our patients, some of them being evidenced with polymyographic recording.

Though pyramidal signs were present in most patients of our series (11/14), two patients only had a significant spasticity. Pyramidal signs are also frequently reported in patients from the literature (6/11, totally 17/25) but spasticity was reported in 3/15 (totally 5/31). Thus, spastic ataxia is not a usual presentation in the phenotypic spectrum of ARCA2.

6/14 patients in our series and 5/17 in the literature had epilepsy (totally 11/31). Seizures were controlled by AEDs or transient in half of the epileptic patients reported here and in other previously reported ones [3,6]. Seizures dramatically worsened in three patients during and after a SLE (patients #5 and #13 and patient 1 in [5]) and was drug-resistant in one patient with infantile encephalopathy (patient #7). Therefore, the severity of epilepsy in ARCA2 is as variable as ataxia, with most patients being mildly affected as long as a SLE does not occur.

Intellectual deficiency (ID) unrelated to a SLE was reported in 7/14 ARCA2 patients of the literature and in 8/14 in our series. It was mild in 9 patients and moderate to severe in 4 (patient #7, [2-6]). It is possible that up to one half of ARCA2 patients have an ID but formal evaluations are obviously lacking, at least in patients with mild ID.

The prominent muscular involvement in patient #12 seems to be unique [7]. Exercise intolerance or muscle weakness was reported in 11/17 patients of the literature. It is not clear, however, whether these patients had acute muscle weakness and myolysis during exercise. The frequency of “true” exercise intolerance or myopathy in ARCA2 still has to be evaluated.

### Paraclinical tests in ARCA2

We report for the first time polymyographic recordings of patients with ARCA2. Our data show that movement disorders in ARCA2 are variable and include subcortical myoclonus, dystonia and tremor. It is worth mentioning that myoclonus in ARCA2 are of low amplitude and less disabling than cortical myoclonus recorded in progressive myoclonic epilepsies, with which ARCA2 shares some features [16,17].

Cerebellar atrophy was present in all reported ARCA2 patients to date and global brain atrophy in two patients with severe involvement (patient #7 and patient 3 in [3]). As discussed above, cerebellar atrophy is expected from the patients of our series screened from an ataxic cohort. The finding of stroke-like lesions on brain MRI in a patient with cerebellar atrophy (like in patients #5 and #13 also reported in [5]) is undoubtedly a clue for the diagnosis of ARCA2. However, stroke-like lesions may attenuate and disappear with time (Figure 1, bottom right).

Since ARCA2 is due to ubiquinone deficiency, mitochondrial disease investigations are particularly expected to provide clues to the diagnosis. Like in our patients series, the concentration of lactic acid was normal in the plasma [4,6] (totally n = 22/27), as well as in the CSF (totally n = 8/9) of most patients. These assays are poorly indicative of ARCA2, except in case of muscular involvement (patient #12, also in [7]). The relevance of ubiquinone assays is conflicting, since it was normal in the fibroblasts of 3/5 patients and low in two others [4,5], low in the blood of one patient and in the muscle of the four patients tested so far [4-6]. From this limited number of patients, muscular level of ubiquinone seems to be the most reliable biological parameter to suggest ARCA2. The assessment of the activity of mitochondrial respiratory chain enzymes suggested CoQ10 deficiency in all analysed muscle samples (patient #7, [2,5-7], but one (patient #8), and in the fibroblasts of some patients [4]. Therefore, when necessary, biological studies performed with muscle samples are undoubtedly the most efficient for the diagnosis of ARCA2.

### Spectrum of *ADCK3* mutations

The p.Arg271Cys and p.Arg299Trp mutations were already reported missense mutations [3]. Since these two missense mutations occur on CpG dinucleotides, which are potential mutation hot-spots, they may represent recurrent mutational events rather than cases resulting from founder events. All other mutations reported here are novel. Two missense mutations alter the highly conserved DFG (Asp-Phe-Gly) motif VII of the kinase domain (p.Phe508Ser) and a highly conserved glycine residue in the C-terminal domain specific of the ADCK3-ADCK4 subfamily (p.Gly615Asp; glycine 615 is conserved in all eukaryotes and in all bacteria having an ADCK3/ADCK4 orthologue). The five other mutations are truncating mutations, including two of them found in intron/exon boundaries (c.1081-1_1082dupGTA and c.589-3C > G).

There is no apparent correlation between mutations and severity of the disease. Indeed, some homozygous truncating mutations (patients #8 and #9) and heterozygous missense associated with a frameshift mutation (patients #3, #4, #5 and #6) can be associated with a relatively mildly progressive disease, while some homozygous missense mutations are associated with a very severe phenotype (patient #7) or a mild phenotype (patients #1 and #2).

### Ubiquinone/CoQ10 supplementation therapy

Because CoQ10 deficiency is the cause of ARCA2, it is logical to anticipate clinical improvement with ubiquinone supplementation therapy with either idebenone or ubidecarenone. Seven ARCA2 patients reported so far received idebenone at different doses ranging from 200 mg to 700 mg per day for adults and 10 mg/kg/day for children. The treatment was evaluated after two months to three years and was considered either inefficient [3], or efficient [4,6], or even harmful [5,7]. Regarding our series, ubiquinone therapy did not lead to significant improvement of the neurological status in most patients. This could be due to the slow clinical course and mild impairment in these patients. Ubidecarenone had reversible side effects in some patients but did not worsen the neurological involvement, in opposition to idebenone in two patients (patient #12 and patient 1 in [5]). Strikingly, movement disorders were markedly improved in two patients reported here under ubidecarenone. Therefore, we would suggest the use of ubidecarenone rather than idebenone but it is obviously too early to definitively conclude on the effectiveness or the ineffectiveness of CoQ10 supplementation in ARCA2 and further study are needed to assess such efficacy. In particular, whether ubidecarenone is efficient in preventing SLE is an important issue without answer to date.

### When to ask for *ADCK3* molecular study?

It appears from the analysis of the 31 ARCA2 patients known to date that the condition encompasses a broad phenotypic spectrum. The ataxia is mostly associated with other neurological signs (movement disorder, pyramidal involvement, epilepsy, ID), rarely with muscular involvement but not with peripheral neuropathy, and is mildly progressive or apparently stable from childhood to adulthood and throughout adulthood. The severity of associated signs seems to be proportionate with that of the ataxia and the phenotype may be aggravated by a SLE in some patients. On the other hand, a minority of patients may have a severe encephalopathy with global brain degeneration and atrophy.

The study of muscular samples from ARCA2 patients proved to be reliable for its diagnosis. Although fully justified given the severe clinical involvement in some patients, as discussed by others [6], it may be regarded as too invasive in most patients with mild disability.

We suggest undergoing first the targeted molecular analysis of *ADCK3* in mildly affected individuals with a suspected ARCA when the phenotype is compatible, including in ataxic patients experiencing a SLE. The diagnosis of other patients with an atypical and/or severe ARCA2 phenotype may also be performed on the basis of biological data, i.e. when low ubiquinone muscular level or functional ubiquinone deficiency is evidenced. Obviously, diagnosis tools using next generation sequencing, such as massively parallel sequencing of targeted genes [18] or exome sequencing [19], which are already or will be soon used for ARCAs, will modify the molecular diagnosis strategy. In these cases, knowledge of the heterogeneous phenotypes associated with mutations in *ADCK3* will be helpful to interpret these results [20].

## Competing interests

The authors declare that they have no competing interests.

## Authors’ contribution

CM, EA, AD, CML, PC, DD, CM, PdL, LB, SCB, ASL, MR, YC, EB, ER, IB, SC, CR, AB, AR, ID, CT, MK and MA designed the study and interpreted the data. ND, NK, EN, SCB, ASL, and MK carried out the molecular genetic studies. CM, EA, AD, MK and MA drafted and revised the manuscript. All authors read and approved the final manuscript.

## Supplementary Material

Additional file 1: Table S1 Clinical, biological, molecular and radiological data from 14 patients with ARCA2. Click here for file

Additional file 2 Clinical, biological, molecular and radiological data from 17 ARCA2 patients of the literature. Click here for file

Additional file 3: Table S2 Detailed description of representative clinical histories. Click here for file
