# Supplementary material for: Phenotypic variability in ARCA2 and identification of a core ataxic phenotype with slow progression
Source: Orphanet J Rare Dis. 2013 Oct 28;8:173. doi: 10.1186/1750-1172-8-173 (PMC3843540; doi:10.1186/1750-1172-8-173)
Supplement: Additional file 1: Table S1 — Clinical, biological, molecular and radiological data from 14 patients with ARCA2. [file 1750-1172-8-173-S1.doc]

Table 1 **Clinical, biological, molecular and radiological data from 14 patients with ARCA2**

|  | **Original report** | | | | | | | | | | Previously reported patients with update^a^ | | | |
| --- | --- | --- | --- | --- | --- | --- | --- | --- | --- | --- | --- | --- | --- | --- |
| **Patient** | #1* | #2* | #3 | #4 | #5* | #6* | #7 | #8* | #9* | #10 | #11 | #12 | #13* | #14* |
| **Origin** | Morocco | Morocco | France | Portugal | France | France | Tunisia | Brazil | Brazil | Brazil | France + Algeria | France | France | France |
| **Sex** | M | F | M | M | F | M | F | F | F | F | F | F | F | F |
| ***ADCK3* mutation** | hmz c.1844G > A exon15 p.Gly615Asp | hmz c.1844G > A exon15 p.Gly615Asp | comp. htz c.1523 T > C exon13 + del exons 3 to 15 p.Phe508Ser | comp. htz c.[895C > T] + [1358delT] exons 7 and 11 p.[Arg299Trp] + [ Leu453Argfs*24] | comp. htz c.[895C > T] + [1228C > T] exons 7 and 10 p.[Arg299Trp] + [Arg410*] | comp. htz c.[895C > T] + [1228C > T] exons 7 and 10 p.[Arg299Trp] + [Arg410*] | hmz c.811C > T exon 6 p.Arg271Cys | hmz c.589-3C > G intron 3 p.Leu197Valfs*20 | hmz c.589-3C > G intron 3 p.Leu197Valfs*20 | hmz c.1081-1_1082dupGTA intron 8 / exon 9 p.Gln360_Tyr361ins* | comp. htz c.[993C > T] + [1645G > A] exons 8 and 14 p.[Lys314_Gln360del] + [Gly549Ser] | comp. htz c.[815G > A] + [1812_1813insG] exons 6 + 15 p.[Gly272Asp] + [Glu605Glyfs*125] | comp. htz c.[637C > T] + [815G > T] exons 4 + 6 p.[Arg213Trp] + [Gly272Val] | comp. htz c.[637C > T] + [815G > T] exons 4 + 6 p.[Arg213Trp] + [Gly272Val] |
| **First signs (age)** | gait ataxia (childhood) | gait ataxia (7y) | writing difficulties, possible dystonia (6y) | gait ataxia + tremor (15y) | gait ataxia + writing difficulties (4y) | writing difficulties, ataxia at examination (4y) | truncal ataxia, delayed motor development, seizures (1.5y) | gait ataxia (19y) | gait ataxia (19y) | tremor, chorea of upper limbs (2y) | gait ataxia + tremor (2-3y) | exercise intolerance (3y) | delayed psychomotor development, clumsy hands (2y) | delayed psychomotor development, clumsy hands (2y) |
| **Disease duration (years)** | 27 | 17 | 16 | 28 | 34 | 30 | 3.5 | 15 | 12 | 13 | 27-28 | 17 | 19 | 13 |
| **Age last examination (years)** | 29 | 24 | 27 | 44 | 38 | 34 | 5 | 34 | 31 | 15 | 29.5 | 20 | 21 | 15 |
| **Ambulatory status** | ambul | ambul | ambul | ambul | ambul | ambul | never walked | ambul | ambul | ambul | ambul | ambul | partial loss after SLE | ambul |
| **Cerebellar ataxia** | yes | yes | yes (onset 10-12y) | yes | yes | yes | yes | yes | yes | yes (onset 7y) | yes | yes (onset 7y) | yes (onset 9y) | yes (onset 5y) |
| **Dysmetria** | yes | yes | yes | yes | yes | yes | yes | yes | yes | yes | yes | yes | yes | yes |
| **Saccadic pursuit^b^** | yes | yes | yes | yes | yes | yes | NA | no | no | no | no | yes | NA | no |
| **Nystagmus** | no | NA | no | no | no | yes | NA | no | yes | no | no | no | yes | no |
| **Early SDFS^c^** | NA | NA | 2 (12y) | 2 (15y) | 2 (8y) | 1 (5y) | 7 (2y) | NA | NA | 3 (4y) | NA | 2 (7y) | 2 (9y) | 1-2 (5y) |
| **SDFSat last examination** | 2 (29y) | 2 (24y) | 2 (26y) | 2 (44y) | 2 (38y) | 2 (since 7.5y, unchanged ay 34y) | 7 (5y) | 3 (34y) | 3 (31y) | 3 (15y) | 3 (29.5y) | 3 (20y) | 5 (18y) then 3-4 (21y) | 1-2 (15y) |
| **SARA score before treatment(age)** | 6 (29y) | 4 (24y) | 9.5 (26y) | 11.5 (45y) | 14 (38y) | 10 (34y) | NA | 7 (34y) | 10 (31y) | 15 (15y) | 13 (29.5y) | 15.5 (20y) | NA | NA |
| **Extrapyramidal signs** | no | no | mild hand dystonia, head tremor (onset 23y) | postural/action tremor of upper limbs 6 Hz | mild dystonia of neck | mild dystonia of hands, mild postural tremor | dystonia, chorea | no | no | transient chorea (from 2 to 7y) | no | mild left upper limb dystonia | right upper limb dystonia | no |
| **Myoclonus (age of onset)** | no | no | yes, neck (23y) | yes, transient | no | no | yes | no | no | no | no | yes, upper limbs (60-135 ms/9y) | yes, during and after SLE | yes |
| **Extensor plantar reflex** | no | no | no | no | no | no | bilateral | no | no | no | no | unilateral | no | no |
| **Tendinous reflexes** | normal | normal | brisk | decreased | brisk | brisk | brisk | very brisk | very brisk | brisk | brisk | brisk | brisk | brisk |
| **Spasticity** | no | no | no | no | no | no | yes | no | no | no | no | no | unilteral after SLE | no |
| **Muscle involvement** | no | no | no | no | no | no | no | no | no | no | no | EI (3y) | no | no |
| **ID^d^** | no | no | no (VIQ 103, PIQ 89, FSIQ 106 at 26y) | no (VIQ 85, PIQ 79, FSIQ 80 at 43y) | mild | mild | severe | no | mild | no | mild (VIQ 61, PIQ 50, FSIQ 54 at 30y) | no | mild (FSIQ 70 at 10y), deteriorated after SLE | no |
| **Early psychomotor development (<3** **years)** | normal | normal | normal | normal | normal, walked at 12 m, normal language | normal, walked at 15 m, normal language | able to sit at 6 m, global regression after 18 m | normal | NA | normal, walked at 14 m | mildly delayed, walked at 14 m, delayed language | normal | mildly delayed, walked at 24 m, first words delayed, hand clumsiness | mildly delayed, walked at 18-20 m, hand clumsiness |
| **Seizures (age of onset), type** | yes, transient P temporal | no | no | yes, 2 GTC seizures at 19 and 20y | yes (19y), GTC | yes (20y), GTC | yes (1.5y), P multifocal | no | yes | no | no | one GTC seizure (3y, no AED) | yes (10y), GTC at onset then SLE then P | no |
| **SLE (age)** | no | no | no | no | yes (34y) | no | no | no | no | no | no | no | yes (14y) | no |
| **EEG** | NA | NA | NA | normal (40y) | bioccipital spikes (32y) | left parieto-occipital spikes (23 y) | altered background activity, multiple foci of spikes and spike-waves | normal background + focal epileptic activity | normal background + focal epileptic activity | normal | normal (26y) | normal | NA | NA |
| **Evidence for progressivity of epilepsy ?** | no, AED withdrawn without relapse | NA | NA | no, AED withdrawn at 25y without relapse | yes, status epilepticus during SLE | no, epilepsy controlled by AED | yes, drug-resistant | NA | no, seizure free since 2y with AED + CoQ | NA | NA | no | yes, seizure-free for 2y, then SLE at 14y with residual epilepsy | NA |
| **AED** | no | no | no | no | sodium valproate | sodium valproate | levetiracetam, clobazam | diazepam and carbamazepine used as mood stabilizer | carbamazepine | no | no | no | levetiracetam, topiramate, clonazepam |  |
| **Plasma lactate** | normal | NA | NA | NA | NA | normal | normal | normal | normal | normal | normal | increased | normal | normal |
| **CSF lactate** | normal | NA | NA | NA | NA | normal | normal | normal | normal | normal | NA | NA | normal | normal |
| **Muscular biopsy** | NA | NA | NA | NA | NA | NA | RRF | normal | normal | normal | NA | RRF and marked increase of mitochondria | NA | NA |
| **Mitochondrial respiratory chain assessment** | NA | NA | NA | NA | NA | NA | reduced complex II + III activity in muscle | normal in muscle | NA | NA | NA | muscle: decresased I + III and II + III activity | NA | NA |
| **CoQ10 level** | NA | NA | NA | normal in fibro | NA | NA | decreased in blood (0,43 μM, N = 1,2 μM) | NA | NA | NA | NA | decreased in muscle, normal in fibro | NA | NA |
| **Brain MRI** | marked vermian atrophy (29y) | cerebellar atrophy (24y) | marked vermian atrophy (26y) | marked vermian atrophy | cerebellar atrophy (8y) | vermian atrophy (12y) | cerebellar atrophy (1.5y) | cerebellar atrophy | cerebellar atrophy | cerebellar atrophy | cerebellar atrophy (29y) | cerebellar atrophy (7.5y) | vermian atrophy (7y) | vermian atrophy |
| **CoQ10 treatment** | ideb. 45 mg X3/day | ideb. 45 mg X3/day | ubid. 100 mg X3/day | ubid. 100 mg X3/day | ubid. 100 mgX3/day | ubid. 100 mgX3/day | ubid. 30 mg/kg/day | ubid. 400 mg X3/day | ubid. 400 mg X3/day | ubid. 400 mg X2/day | no | ubid. 250 mg X3/day (worsening with ideb.) | ubid. 175 mg X2/day | ubid. 175 mg X2/day |
| **Duration of CoQ10 treatment** | 9 m | NA | 15 m | 8 m | 1 m | 1 m | 3y | 12 m | 12 m | 12 m | NA | 15y | 8y | 13 m |
| **Evaluation of therapy** | NA | NA | impr. of mov. disord., SARA 6.5 | impr. of mov. disord. | withdrawn, reversible side effect (anorexia) | withdrawn, reversible adverse effect (diarrhea) | no clear impr. | no clear impr., SARA 7/40 unchanged | no clear impr., SARA 9/40 | no clear impr. | NA | no clear impr. | no clear impr. | no clear impr., withdrawn |

Abbreviations: origin: geographical origin; sex: M male, F female; hmz: homozygous; comp. htz: compound heterozygous; y: years; m: months; NA: not available or not applicable; ambul: ambulatory; SLE: stroke-like episode; ID: intellectual deficiency; VIQ: verbal intelligence quotient; PIQ: performance intelligence quotient; FSIQ: full scale intelligence quotient; seizure type: P partial, GTC generalized tonic clonic; CSF: cerebrospinal fluid; CoQ10: coenzyme Q10 = ubiquinone; AED: anti-epileptic drugs; RRF: ragged-red fibers; fibro: skin fibroblasts; ideb.: idebenone, ubid.: ubidecarenone; impr.: improvement; MRI: magnetic resonance imaging; TDM: tomodensotimetry; SD: standard deviations

^*^ Successive patients marked with “*” belong to the same sibship.

^a^References: patient #11 was previously reported in [4] (as patient 7); patient #12 in [5] and in [7] (as patient 4); patient #13 in [5] (as patient 2); patient #14 in [5] (as patient 3).

^b^refers to smooth pursuit eye movements

^c^SDFS: 0: no functional handicap ; 1: no functional handicap but signs at examination ; 2: mild, able to run ; 3: moderate, unable to run ; 4: severe, walking with one stick, walking unlimited ; 5: walking with two sticks ; 6: unable to walk, requiring wheelchair, limited walking without aid ; 7: confined to bed (idem). Early SDFS: SDFS early in the disease course; it was rated with either the pediatric medical record of the patient or with the patient himself and his parents.

^d^The IQ was formally evaluated with the Wechsler adult intelligence scale (WAIS III) for patients #3, #4 and #1, and with the WISC: Wechsler intelligence scale for children (WISC IV) for patient #13.

^e^See Additional file for details.
