# Supplementary material for: Phenotypic variability in ARCA2 and identification of a core ataxic phenotype with slow progression
Source: Orphanet J Rare Dis. 2013 Oct 28;8:173. doi: 10.1186/1750-1172-8-173 (PMC3843540; doi:10.1186/1750-1172-8-173)
Supplement: Additional file 2 — Clinical, biological, molecular and radiological data from 17 ARCA2 patients of the literature. [file 1750-1172-8-173-S2.pdf]

Table 1. Clinical, biological, molecular and radiological data from 17 ARCA2 patients of the literature.

**Reference**	Terracciano et al.	Gerards et al.					Lagier-Tourenne Am J Hum Genet 2008						Mollet et al.	Horvath et al.			
**Patient**	CHA987	A IV:1	A IV:2	A IV:4	B II:1	B II:4	Fam 1 Pat 1	Fam 1 Pat 2	Fam 1 Pat 3	Fam 1 Pat 4	Fam 2 Pat 5	Fam 3 Pat 6	Pat 1	Pat 1	Pat 2	Pat 3	Pat 4
**Geo origin**	NA	Dutch			NA		Algeria				Algeria	USA	Reunion Island	England	Germany	Norway	Kosovo
**ADCK3 mutation**	hmz c.1042C > T p.R348X	hmz c.1042C>T p.R348X exon 8			htz c.1042C>T, p.R348X (exon 8), c.1136T>A, p.Leu379X (exon 9)		hmz c.1398+2T/C exon11 p.[Asp420Trp fsX40,Ile467AlafsX22]				htz c.500_521delinsTTG exon 3 p.Gln167LeufsX36	htz c.[1541A/G] + [1750_1752 delACC] exons 13 and 15 p.[Tyr514Cys] + [Thr584 del]	hmz c.1655G>A, exon 14 p.E551K	htz c.811C>T, p.Arg271Cys, c.910G>A, p.Ala304Thr	hmz c.911C>T, p.304Ala>Val	hmz c.895C>T, p.Arg299Trp	? c.1286A>G, p.Tyr429Cyr htz, second mut not found
**Age at onset (years)**	6	3	9	3	2	#2	11	4	7	8	4	5	1-2	15	27 or childhood	3	1.5

**First signs**	epilepsy	gait ataxia	incoordination, speech diff, gait ataxia	"disturbed motor development"	gait ataxia, dysarthric speech, tremor	gait ataxia, dysarthric speech	gait ataxia	gait ataxia	gait ataxia	gait ataxia	NA	NA	gait ataxia	gait ataxia, writing difficulties	epilepsy	epilepsy (tonic-clonic and absence seizures)	gait ataxia then global developmental slowing
**Age last examination (years)**	17	31	26	25	20	21	42	38	36	29	18	17	16	46	>50	16	25
**Disease duration (years)**	11	28	17	22	18	19	31	34	29	21	14	12	14-15	31	>30	13	23.5
**Early psychomotor development (<3y)**	normal	normal	normal	normal	normal until 2y, then ataxia	normal until 2y, then ataxia	NA	NA	NA	NA	NA	NA	delayed from 1y	normal	normal	normal, walked at 16m	motor (18m) then global regression
**Ambulatory status**	ambul (17y)	ambul (31y)	ambul (26y)	ambul (25y)	ambul (20y)	ambul (21y)	ambul (42y)	ambul (38y)	ambul (36y)	ambul (29y)	ambul (18y)	ambul (17y)	wheelchair bound (13y)	ambul (46y)	ambul (>50y)	wheelchair bound (12y)	wheelchair bound (<20y)
**Evidence for progressiv**	yes, from 6 to 13y	unclear	yes, from 9 to 13, then stable	unclear	no, ataxia improve	no	NA	NA	NA	NA	no	NA	yes, after SLE	from 15 to 18yo	no	yes	yes

**ity?**					d												
**Cerebellar ataxia**	yes, onset >6y	yes, onset 3y	yes, onset 9y	yes, onset 3y	yes, onset 2y	yes, onset 2y	yes, onset 11y	yes, onset 4y	yes, onset 7y	yes, onset 8y	yes, onset 4y	yes, onset 5y	yes, from first steps (1y)	yes (onset 15y)	yes (first noticed at 46y)	yes (obvious at 3y)	yes (onset in childhood)
**Intention tremor**	yes, onset 6y	yes at 31y	yes at 26y	yes at 25y	NA	NA	NA	NA	NA	NA	NA	NA	NA	yes	yes	yes	yes
**Dysarthric speech**	yes, onset 6y	NA	yes, onset 9y	yes (25y)	NA	NA	NA	NA	NA	NA	NA	NA	NA	yes	yes	yes	yes
**Smooth pursuit[a]**	NA	saccadic	saccadic	NA	NA	NA	NA	NA	NA	NA	NA	NA	NA	slow	normal	NA	saccadic
**Nystagmus**	NA	NA	NA	NA	NA	NA	NA	NA	NA	NA	NA	NA	NA	NA	no	NA	yes
**SDFS**	NA	NA	NA	NA	NA	NA	3	3	3	3	3	3	NA	NA	NA	NA	NA
**Extensor plantar reflex**	NA	NA	no	NA	NA	no	no	no	no	yes	no	yes	NA	NA	NA	NA	yes
**Tendinous reflexes**	NA	NA	brisk	NA	NA	normal	absent ankles	normal	brisk	brisk	normal	brisk	NA	normal	brisk	NA	brisk
**Spasticity**	no	no	no	no	mild	no	no	no	no	no	no	no	NA	NA	yes	no	spastic tetraparesis (25y)

**Muscle involvement (age of onset)**	NA	EI (20y)	EI (26y)	EI (19y)	EI (>20y)	EI	no	EI	EI	EI	no	NA	NA	mild EI	muscle weakness	muscle weakness	NA
**Other motor involvement**	no	dystonic posture (31y), swallowing difficulties (20y)	no	no	mild dystonic movements (>20y)	no	no	no	no	no	no	mild dystonia of hands and feet	NA	mild swallowing diff., mild dystonic posturing, tremor	tremor	swallowing diff. requiring a gastrostomy (13y)	no
**Myoclonus (age of onset)**	NA	yes (20y)	no	NA	no	no	NA	NA	NA	NA	NA	NA	NA	yes	yes (27y)	no	no
**Epilepsy (age of onset)**	yes (6y) partial seizures	no, abnormal EEG	no	yes (19y)	no	no	no	no	no	no	no	no	yes (2.5y)	no	yes (childhood)	yes (3y)	no
**SLE (age)**	no	no	no	no	no	no	no	no	no	no	no	no	yes (12y)	no	no	no	no
**ID (age)**	mild (8y)	NA	mild (26y)	NA	no	no	no	mild (8y)	no	no	mild (18y)	no	sev. (12y)	NA	no	mod./sev. progressive	mod./sev.
**Plasma lactate**	normal	NA	NA	NA	NA	NA	NA	3.3 (n=0.5-	2.9 (n=0.5-	1.8-7.8 (n=0.5-	1.29 (n=0.5-2.2)	0.7 (n=0.5-2.2)	3 (12y during	NA	NA	NA	NA

**(mM)**								2.2)	2.2)	2.2)			SLE; n=1-1.55)				
**CSF lactate (mM)**	NA	NA	NA	NA	NA	NA	NA	NA	NA	NA	NA	NA	4 mM (2.5y)	NA	NA	NA	NA
**Muscular biopsy**	8y	NA	NA	NA	at 10 and 26y, normal morphology	NA	NA	NA	NA	NA	NA	NA	NA	hyperreactive SDH staining, some negative Cox staining fibres, no RRF	hyperreactive SDH staining, some negative Cox staining fibres, no RRF, increased lipid accumulation		hyperreactive SDH staining, some negative Cox staining fibres, no RRF, increased lipid accumulation
**Mitochondrial respiratory chain assessmen**	↓ act. of cx I+III and II + III in muscle	NA	NA	NA	NA	↓ act. of cx II + III to 23% of control	NA	NA	NA	NA	NA	NA	↓ act. of cx II+III in muscle				

**t**						values in muscle											
**CoQ10 level**	low in muscle	NA	NA	NA	NA	NA	NA	NA	normal in fibro	NA	low in fibro	low in muscle and in fibro	low in muscle, normal in fibro	NA	NA	NA	NA
**CoQ10 treatment**	ideb. 10 mg/kg/d (8-14y), clear improvement of ataxia within 6m	no	no	no	no	no	no	no	no	no	no	no	ideb. 5 then 10 mg/kg/day from 3y 8m to 7y, no effect	ideb. 300 mg/d during 6m, no effect	ideb. 300 mg/d during 6m, no effect	ideb. 200 mg/d during 2m, no benefit	ideb. 200 mg/d during 2m, no benefit
**Brain MRI/TDM (age)**	mild CA at 7y (MRI), worsened at 13y	CA (31y) (MRI)	CA (TDM)	CA (26y) (MRI)	CA, (stable from 16 to 26y)	CA, (stable from 11 to 21y)	CA	CA	CA	CA	CA	CA	severe CA + stroke-like lesions (12y)	CA	prominent CA	progressive CA	prominent CA

Abbreviations: geo origin: geographical origin; hmz: homozygous; htz: compound heterozygous; y: years; m: months; SLE: stroke-like episode; ID: intellectual deficiency; mod.: moderate; sev.: severe; diff.: difficulties; SDH: succinate dehydrogenase; RRF: ragged red fibres; act.: activity; cx: mitochondrial complex; CoQ10: coenzyme Q10; fibro: skin fibroblasts; ideb.: idebenone; MRI: magnetic resonance imaging; TDM: tomodensitometry; CA: cerebellar atrophy

[a]smooth pursuit eye movements
