# Supplementary material for: Phenotypic variability in ARCA2 and identification of a core ataxic phenotype with slow progression
Source: Orphanet J Rare Dis. 2013 Oct 28;8:173. doi: 10.1186/1750-1172-8-173 (PMC3843540; doi:10.1186/1750-1172-8-173)
Supplement: Additional file 3: Table S2 — Detailed description of representative clinical histories. [file 1750-1172-8-173-S3.pdf]

SUPPLEMENTARY TEXT

Detailed description of representative clinical histories

Patient #3 was the second child of healthy parents and had normal initial psychomotor development. He reports no motor signs until the age of 6 years when his writing became slow and clumsy. These writing difficulties remained unchanged and were retrospectively consistent with mild hand dystonia. Obvious gait ataxia appeared at the age of 12 years, preventing him from riding a bicycle. Since then, gait imbalance did not clearly worsen. Patient #3 had no history of seizures or exercise intolerance but an intermittent neck tremor from 23 year-old on. When first examined at 26 years, his cerebellar syndrome was mild (SARA 9.5/40) and associated with mild hand dystonia and a writer cramp, subtle myoclonic jerks, intermittent head tremor and brisk tendon reflexes without spasticity. He had no limitation in walking and was able to run (SDFS 2/7). Polygraphic EMG recording evidenced myoclonus in neck muscles and upper limbs and provided a diagnosis of dystonic tremor of the neck (Fig. 2A). Brain MRI disclosed marked vermian atrophy. Neuropsychological evaluation confirmed a normal intellect (WAIS-III, VIQ 103, PIQ 89, FSIQ 106).

From the age of 26 years on, he received a trial of ubidecarenon (100 mg x3/d) and was reevaluated after 6 months of treatment. He reported a clear improvement of tremor. Clinical examination found mild improvement of the cerebellar syndrome (SARA 6/40) and a disappearance of head tremor and myoclonus, which was confirmed by polymyographic recording (Fig. 2B).

Patient #5 is the sister of **patient #6**. They both have similar clinical history until adulthood. Pregnancy, delivery and psychomotor development were unremarkable.

They walked independently at or before the age of 15 months. First signs were noticed at 4 year-old and were characterized by slow writing. When first examined by a neuropediatrician at 8.5 year-old, the parents of patient #5 reported an ataxic gait that started at 4 years and that seemed to improve with time because the child had fewer falls. She had mild cerebellar signs with little handicap (SDFS 2/7) but a clear writing limitation. When his brother was first examined at the age of 5, he also had mild cerebellar signs (SDFS 2/7) and writing difficulties. Brain MRI showed cerebellar atrophy in both children. The neurological follow-up until the age of 24 years (patient #5) and 20 years (patient #6) did not reveal any obvious worsening of ataxia. Both patients needed special education because of motor difficulties. Both had generalized seizures at 19-20 year-old and were treated with sodium valproate.

Patient #6, now aged 34 years, had only one seizure since then. He is able to walk without support and is able to run (SDFS 2/7). Clinical examination disclosed a moderate cerebellar syndrome with ataxia, gaze-evoked nystagmus (SARA 10/40), hand tremor with mild hand dystonia, and brisk tendon reflexes but no spasticity.

At the age of 35 years, patient #5 underwent gastric sleeve surgery and lost 20-30 kg of body weight. Afterwards, she had to be hospitalized during a subacute episode of headache and visual loss followed by status epilepticus. Brain MRI revealed unilateral occipital and parietal lesions compatible with a stroke-like episode (Fig. 1, bottom right). She is presently 38 year-old and still has visual impairment and mild gait difficulties (SDFS 2/7). At examination, the cerebellar syndrome is moderate (SARA 14/40) with hypermetric eye saccades and brisk tendon reflexes without spasticity.

Patient #7 was the first child of related parents of Tunisian origin. She was a full term neonate with low birth weight (2515 g), height (45 cm) and occipitofrontal circumference (31.8 cm). Her psychomotor development was reportedly normal until the age of 15 months when she had a first febrile seizure. When hospitalized at the age of 17 months because of status epilepticus, marked truncal hypotonia and delayed acquisitions were noticed. She was able to grab objects, to sit alone and said a few words. Brain MRI showed cerebellar atrophy. Three months later, she had lost her ability to sit up and her language regressed. Cerebellar signs were evidenced but she had no pyramidal or extrapyramidal signs. At 22 months, she underwent a muscle biopsy that identified an oxidative phosphorylation defect compatible with CoQ10 deficiency, as well as ragged-red fibers. More precisely, spectrophotometric assessment of oxidative phosphorylation enzyme activities in mitochondria isolated from muscle revealed a normal complex IV activity and reduced complex II+III activity (154 nmol/min/mg protein, N=235 ±62). The adjunction of ubiquinone increased the enzymatic activity of complex II+III by 192.1% (N=125 ±6.5), indicating partial ubiquinone deficiency. This result combined with low plasma ubiquinone concentration (0,43 µM, N=1,2 µM) induced molecular study of the *ADCK3* gene revealing the homozygous p.Arg271Cys mutation.

At 22 months, patient #7 had lost her ability to grab objects and her ocular contact became weak. During the following years, her neurological status worsened despite a trial of ubidecarenon (300 mg x3/d). Spastic tetraparesis, myoclonus, dystonia and chorea were present at examination at the age of 5 years. Her epilepsy became drug-resistant and microcephaly became obvious (-3.5 standard deviations at 4 years), together with global cerebral atrophy.
